# Scientific Items

**Published:** 1884-10-20

**Authors:** 


### SCIENTIFIC ITEMS.

**The Banyan Tree.**—Forbes, in his Oriental Memoirs, says that a banyan tree, named Cubbeer Burr, was nearly two thousand feet in circumference, measured round its principal stems, but that the ground covered by its overhanging branches was considerably more extensive. The large trunks numbered three hundred and fifty, and the smaller ones exceeded three thousand. This tree, at one time, was considerably larger, a fearful storm, accompanied by a flood on the Nerbudda, having carried away a greater part of it, reducing the number of the larger trunks from thirteen hundred and fifty to the three hundred and fifty now remaining. The original size of this colossal tree may be better conceived by remembering that two thousand feet (its circumference when Forbes saw it) is more than one-third of a mile. It is truly one of the wonders of Nature. The careful provision by which everything is made to adapt itself to the circumstances in which it is placed is strongly exemplified in the growth of this tree; for if these branches did not throw out roots, and so form a trunk with which to support their own weight, they would tear themselves off from the parent stem.—*Pop. Sci. News.*

**A Paper Door.**—"Feel the weight of that door," said a New York builder to a reporter, who was looking at an unfinished apartment house up-town. The reporter prepared to lift what seemed a polished mahogany door, but it proved too light for any wood. "It is made of paper," said the builder, "and, while it costs about the same as wood, is much better, because there is no shrinking, swelling, cracking or warping. It is composed of two thick paper boards stamped and moulded into panels and glued together with glue and potash, and then rolled through heavy rollers. It is first covered with a water-proof coating, and is painted and varnished and hung in the ordinary way. Few persons can detect that they are not made of wood, particularly when used as sliding doors."—*Mech. News.*

**A Curious Calculation.**— In a recent lecture "On Fixed Stars," Dr. David Gill wanted an illustration of the distance to Centauri. This is what he said: "We are a commercial people. We like to make our estimates in pounds sterling. We shall suppose that some wealthy directors have failed in getting parliamentary sanction to cut a sub-Atlantic tunnel to America, and so, for want of some other outlet for their energy and capital, they construct a railway to Centauri. We shall neglect, for the present, the engineering difficulties—a mere detail—and suppose them overcome and the railroad open for traffic. We shall go further, and suppose that the directors have found the construction of such a railway to have been peculiarly easy, and that the proprietors of the interstellar space had not been exhorbitant in their terms for right of way. Therefore, with a view to encourage traffic, the directors had made the fares exceedingly moderate, viz.: first-class at one penny per one hundred miles. Desiring to take advantage of these facilities, an American gentleman, by way of providing himself with small change for the journey, buys up the national debt of England and of a few other countries, and, presenting himself at the booking office, demands a first-class single to Centauri. For this he tenders in payment the scrip of the national debt of England, which just covers the cost of his ticket; but I should explain that at this time the national debt, from little wars, coupled with some unremunerative government investments in landed property, had run up from seven hundred millions of pounds to one billion, one hundred millions of pounds sterling. Having taken his seat, it occurs to him to ask: 'At what rate do you travel?' 'Sixty miles an hour, sir, including stoppages,' is the answer. 'Then, when shall we reach Centauri?' 'In forty-eight million, six hundred and sixty-three thousand years, sir.' 'Humph! rather a long journey.'"—*Boston Advertiser.*

**Electricity in Dental Practice.**—So pure and so intense are the rays derived from electricity that, even with the imperfect apparatus already devised, a sight of the oral tissues under its illumination is a new revelation to the dentist. We do not mean to assert that the teeth and their investing tissues are made transparent, but we do say that so intense is the illumination that the condition of the tissues can be, by the experienced operator, very clearly observed. The congestion produced by pressure alters the appearance of the soft tissues, so that, when comparatively deep-seated, it is quite plainly indicated. An inflamed pulp changes the looks of a tooth, interrupting the semi-translucency. A filling can be plainly seen through a tooth, and a filled root presents a different appearance from one that is unfilled. But it is especially in determining the condition of proximate surfaces in close contact that the usefulness of the electric light becomes evident. If such surfaces are rough and unpolished, if food habitually lodges between them, the fact is at once apparent. If softening of the enamel has begun, the premonitory symptom of approaching decay, it is instantaneously revealed. If a pocket has formed beneath the gum and foreign matter is lodged there, it needs not the probe to determine the fact. Incipient pyorrhœa can be immediately detected.

The apparatus with which these results have been obtained is by no means perfect. When something a little better shall have been offered, we may confidently look to see electricity employed to advantage in the office of every operating dentist.—*Med. Times.*

**Electric Ballet.**—London is to have an electric ballet. The stage will be darkened for a moment only to be instaneously illuminated with hundreds of ballet-girls in armor, and every point of it pricked with stars of electric light. An ingenious Frenchman is the author of this device. When he arrived at the Charing-Cross station with his ballet dresses and machinery, he was promptly arrested as a Parisian Fenian, and had to prove his connection with the theatrical trade before he was released.

